# Effectiveness of calf muscle stretching for the short-term treatment of plantar heel pain: a randomised trial

**DOI:** 10.1186/1471-2474-8-36

**Published:** 2007-04-19

**Authors:** Joel A Radford, Karl B Landorf, Rachelle Buchbinder, Catherine Cook

**Affiliations:** 1 School of Biomedical and Health Sciences, University of Western Sydney, Locked Bag 1797, Penrith South DC, NSW, 1797, Australia; 2 Department of Podiatry, School of Human Biosciences, La Trobe University, Bundoora, Victoria, 3086, Australia; 3 Monash Department of Clinical Epidemiology at Cabrini Hospital and Department of Epidemiology and Preventive Medicine, Monash University, Malvern, Victoria, 3144, Australia

## Abstract

**Background:**

Plantar heel pain is one of the most common musculoskeletal disorders of the foot and ankle. Treatment of the condition is usually conservative, however the effectiveness of many treatments frequently used in clinical practice, including stretching, has not been established. We performed a participant-blinded randomised trial to assess the effectiveness of calf muscle stretching, a commonly used short-term treatment for plantar heel pain.

**Methods:**

Ninety-two participants with plantar heel pain were recruited from the general public between April and June 2005. Participants were randomly allocated to an intervention group that were prescribed calf muscle stretches and sham ultrasound (n = 46) or a control group who received sham ultrasound alone (n = 46). The intervention period was two weeks. No participants were lost to follow-up. Primary outcome measures were 'first-step' pain (measured on a 100 mm Visual Analogue Scale) and the Foot Health Status Questionnaire domains of foot pain, foot function and general foot health.

**Results:**

Both treatment groups improved over the two week period of follow-up but there were no statistically significant differences in improvement between groups for any of the measured outcomes. For example, the mean improvement for 'first-step' pain (0–100 mm) was -19.8 mm in the stretching group and -13.2 mm in the control group (adjusted mean difference between groups -7.9 mm; 95% CI -18.3 to 2.6). For foot function (0–100 scale), the stretching group improved 16.2 points and the control group improved 8.3 points (adjusted mean difference between groups 7.3; 95% CI -0.1 to 14.8). Ten participants in the stretching group experienced an adverse event, however most events were mild to moderate and short-lived.

**Conclusion:**

When used for the short-term treatment of plantar heel pain, a two-week stretching program provides no statistically significant benefit in 'first-step' pain, foot pain, foot function or general foot health compared to not stretching.

## Background

Plantar heel pain (plantar fasciitis) can be a painful and debilitating condition. It is highly prevalent with one recent United States study estimating that one million patient visits each year at office-based physicians and hospital outpatient departments are for the diagnosis and treatment of plantar heel pain [1]. The disorder appears in sedentary populations [2-4] with seven percent of adults aged 65 years or older found to have plantar heel pain [2,3]. It also makes up one quarter of all foot injuries in runners [5] and up to 8% of all injuries to people participating in sporting activities [6-8]. It is the third most common running injury behind patellofemoral pain and iliotibial band friction syndrome [9]. The condition is thought to be multifactorial in origin with factors such as increased age, decreased ankle and first metatarsophalangeal joint range of motion, obesity and excessive periods of weight-bearing activity commonly suggested to be involved [10,11].

A wide variety of management strategies have been developed to treat the disorder. A systematic review [12] identified 26 different conservative treatments that have been recommended for the treatment of plantar heel pain. At the time of the review, only heel pads, orthoses, steroid injections, night splints and extracorporeal shock wave therapy had been evaluated in randomised trials. The review found that although there is limited evidence for the effectiveness of local corticosteroid therapy, the effectiveness of other frequently employed treatments in altering the clinical course of plantar heel pain had not been established.

One of the more common conservative treatments for plantar heel pain is foot orthoses [13], however due to the manufacturing process there is often a period of a few weeks between the initial consultation and issuing the devices. As such, short-term treatments such as muscle stretches are regularly used to alleviate symptoms during this interim period. A recent systematic review [14] of randomised trials examined the effect of calf muscle stretching on ankle range of motion and found that stretching produces a small but statistically significant increase in ankle range of motion. Such an increase may reduce the symptoms of plantar heel pain by reducing the strain in the plantar fascia that the calf muscle places on it during standing and ambulation [15,16]. However, it is unclear whether a change in ankle range of motion translates to a clinically relevant outcome for patients.

There have been no randomised controlled trials that have examined the effectiveness of calf muscle stretching per se. Two previous randomised controlled trials have compared two active stretching interventions for plantar heel pain: calf stretching compared with plantar fascia stretching [17] and sustained calf stretching compared with intermittent stretching [18]. Neither trial included a sham or no treatment control group, so the effect of calf muscle stretching by itself has not been examined. We conducted a randomised sham-controlled trial to determine whether calf muscle stretching is an effective short-term treatment for plantar heel pain.

## Methods

A randomised, participant-blinded trial was conducted between April and June 2005. Participants with a clinical diagnosis of plantar heel pain and who provided informed consent were randomly allocated to one of two groups: (i) an intervention group receiving calf muscle stretching with sham ultrasound, or (ii) a control group receiving sham ultrasound only. Participants were informed prior to entering the study that a sham intervention was being administered in the trial and were blinded as to whether they received active treatment (i.e. stretching) or not. Ethical approval for the trial was gained from the University of Western Sydney Human Research Ethics Committee.

### Participants

Participants were included if diagnosed with plantar heel pain defined as (i) localised pain at the plantar heel; (ii) that was worst when first standing or walking after rest; and (iii) that improved initially after first standing but worsened with increasing activity. As plantar heel pain is diagnosed clinically the majority of the time, we chose to not use expensive imaging procedures for diagnosis; thus maximising generalisability of our findings to standard clinical practice. Participants also needed to be 18 years of age or older and have had symptoms for four weeks or longer. Patients were excluded from the trial if patient history revealed any inflammatory, osseous, metabolic or neurological abnormalities. They were also excluded if they had received a corticosteroid injection within the past three months. Participants were encouraged not to commence use of any new treatments during the trial (e.g. anti-inflammatory medication, foot orthoses etc.).

### Clinical protocol

Participants were recruited from local community newspaper advertisements in Campbelltown (Sydney, Australia) and treated at a university podiatry clinic. The random allocation sequence was generated using a computer program (Microsoft Excel) in one block (i.e. simple randomisation). The allocation sequence was concealed from the researcher (JR) enrolling and assessing participants in sequentially numbered, opaque, sealed and stapled envelopes. Aluminium foil inside the envelope was used to render the envelope impermeable to intense light. To prevent subversion of the allocation sequence, the name and date of birth of the participant was written on the envelope and a video tape made of the sealed envelope with participant details visible. Carbon paper inside the envelope transferred the information onto the allocation card inside the envelope and a second researcher (CC) later viewed video tapes to ensure envelopes were still sealed when participants' names were written on them. Corresponding envelopes were opened only after the enrolled participants completed all baseline assessments and it was time to allocate the intervention.

Three minutes of *sham *ultrasound (Accusonic AS250, Metron) was then given to the painful heel regardless of whether participants had been allocated the active intervention (i.e. stretching) or not. The ultrasound unit was powered with all operational lights activated. At commencement of treatment the researcher (JR) increased the wattage of the machine which was accompanied by 'beeping' sounds. However, no ultrasound was emitted as the internal timer was not activated (an external timer was used instead).

**Figure 1:**
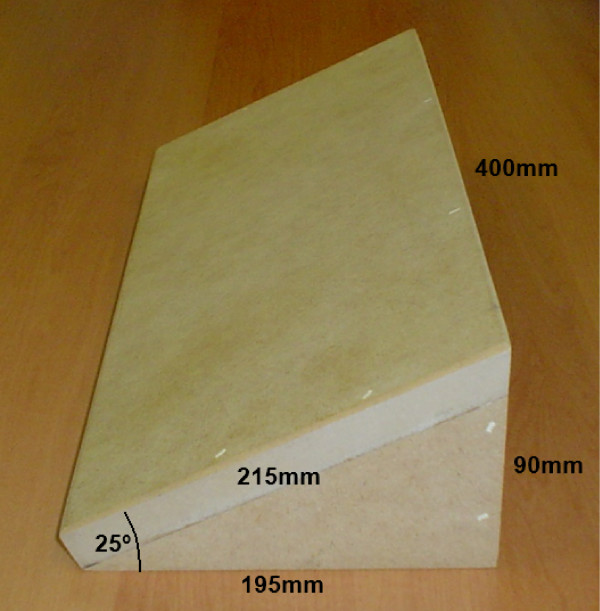
Stretching wedge.

Participants in the stretching group were then given a wooden stretching wedge (Figure 1) on which to perform all stretches. This wedge was used to standardise the stretching technique across participants. The stretching technique was to be performed while standing. Participants were instructed to move their forefoot up the wedge until a stretch could be felt in the calf muscle while keeping their heel on the ground. They were advised to stretch the muscle for at least 5 minutes a day (a daily journal of their stretching was kept by all participants). They were permitted to stretch in smaller sessions (e.g. 1 minute) as long as a total of at least 5 minutes a day was achieved. Participants were warned not to overstretch the muscle and to reduce the force of the stretch by lowering their foot down the wedge if pain was felt in the calf muscle while stretching. Advice was given to stretch every day until the follow-up appointment 14 days later. Participants were not given any further instruction until the end of the trial. To assist with blinding, participants exited the building by a different doorway to the one through which they entered, thus minimising the likelihood of contact with other trial participants.

Outcome assessment was performed at baseline and 14 days. Baseline variables that were collected included age, sex, weight, self-reported hours on feet and duration of symptoms. Primary outcome measures were 'first-step' pain – the pain experienced when first standing after arising from bed in the morning – measured on a 100 mm Visual Analogue Scale and the Foot Health Status Questionnaire which has four domains covering foot pain, foot function, footwear and general foot health (although we pre-specified that we would not analyse the footwear domain). The Foot Health Status Questionnaire has been previously validated (content, criterion and construct validity) across a wide spectrum of pathologies including skin, nail and musculoskeletal disorders. It has a high test-retest reliability (intraclass correlation coefficients ranging from 0.74 to 0.92) and a high degree of internal consistency (Cronbach's α ranging from 0.85 to 0.88) [19]. Both measures are self-administered; however to minimise the investigator having influence on participant responses, participant-completed outcome assessments were performed prior to each consultation.

Secondary outcome measures were ankle range of motion and foot posture. Weight-bearing ankle dorsiflexion (a measure of ankle joint tightness) was measured using the Lunge Test [20] which is reported to also have high intra-rater (intraclass correlation coefficient 0.98) and inter-rater reliability (intraclass correlation coefficient 0.97). Foot posture (e.g. whether someone has a low- or high-arched foot) was measured using the Foot Posture Index (FPI-6) which is reported to have high internal consistency (Cronbach's α 0.83) and high test-retest reliability (intraclass correlation coefficients ranging from 0.62 to 0.91) [21]. The researcher completing follow-up measurement of ankle dorsiflexion and foot posture was not blinded to participant allocation.

### Sample size, data handling and analysis

The sample size of 92 (i.e. 46 per group), calculated *a priori*, was based upon the ability to detect a minimal important difference of 10 mm [22-24] on the Visual Analogue Scale (standard deviation 17) between groups with 80% probability and alpha level of 0.05. This sample size also provided adequate power to detect a minimal important difference between groups of 12 points on the pain domain of the Foot Health Status Questionnaire (standard deviation 20). We conservatively ignored the extra precision provided by the covariate analysis when estimating sample size.

Independent researchers (not otherwise involved in the trial – see acknowledgments) performed data entry and were blinded to group allocation. Double data entry was used to check for errors. Statistical analyses were performed while researchers were blinded to group allocation.

An independent sample t-test was used to determine if there was a difference between groups in the number of days between baseline and follow-up appointments. Outcome data were analysed by intention to treat and according to a pre-planned protocol (i.e. *a priori*). To maximise precision of estimates, analysis of covariance (ANCOVA) was conducted using a linear regression approach [25,26]. The primary outcomes analysed were the change in 'first-step' pain (Visual Analogue Scale), foot pain, foot function and general foot health (Foot Health Status Questionnaire). Secondary outcomes included ankle range of motion (Lunge Test) and foot posture (FPI-6). We pre-specified that the *baseline outcome measure *would be used as the only covariate in each analysis [27] therefore for each of the primary and secondary outcomes we adjusted for the outcome at baseline.

Participants were also asked which intervention (active, sham or don't know) they thought they had received and an index [28] calculated to assess the success of blinding. The index takes the value 1 for complete blinding and 0 for complete lack of blinding; 0.5 is the equivalent of random guessing.

## Results

**Figure 2:**
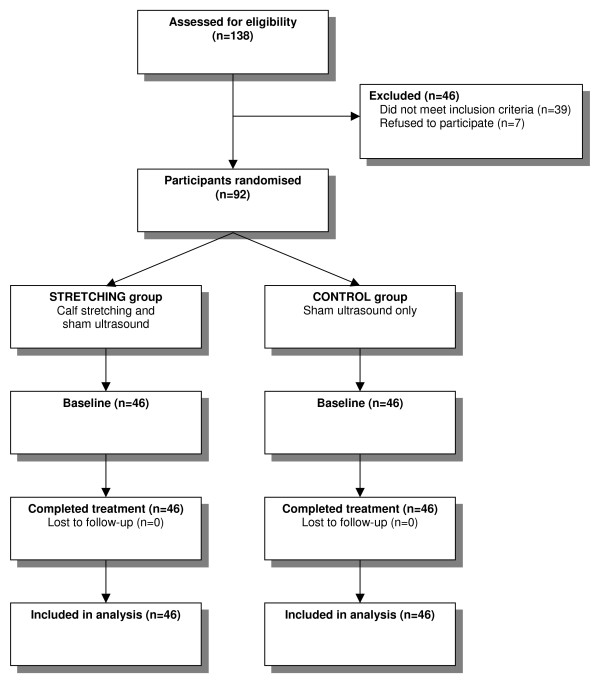
Participant flow diagram.

The flow of participants through the trial is shown in Figure 2. There were no participants lost to follow-up. Baseline characteristics (Table 1) of both groups were similar although the control group had less women 25 (54%) versus 31 (67%) and were on their feet slightly longer each day (mean 9.1 SD ± 3.7 hours versus mean 7.5 SD ± 3.5 hours).

Participants in the stretching group stretched for a median of 14 days (range 5 to 16 days). There was no difference between the groups in time to follow-up (p = 0.489). The median time between baseline and the review appointment was 14 days (range 13 to 16) for the stretching group and 14 days (range 13 to 15) for the control group.

Both the stretching and control group improved in 'first-step' pain, foot pain, foot function and general foot health over the two weeks of follow-up. When compared to the control group, the stretching group demonstrated a small improvement in 'first-step' pain (ANCOVA-adjusted mean difference between groups: -7.9 mm; 95% CI -18.3 to 2.6) and foot function (ANCOVA-adjusted mean difference between groups: 7.3; 95% CI -0.1 to 14.8) but these were not statistically significant. Similarly there were no statistically significant differences between groups for any of the other primary or secondary outcomes (Tables 2 and 3).

Ten participants (22%) in the stretching group experienced adverse events: increased heel pain while stretching (n = 4), calf pain (n = 4), and a new pain in lower limb (n = 2). Adverse events were recorded as mild (n = 4), moderate (n = 2) or severe (n = 4) in nature. One participant discontinued treatment after 5 days of stretching due to severe heel pain while stretching. Upon cessation of stretching, all adverse events resolved. There were no adverse events reported in the control group.

With respect to blinding, thirty-six participants (78%) in the stretching group correctly identified their treatment group compared with twelve participants (26%) in the control group. Seven participants (15%) in the stretching group were uncertain which treatment they received, compared with twelve participants (26%) in the control group. Three participants (7%) in the stretching group and twenty-two participants (48%) in the control group incorrectly identified their treatment group. The blinding index was 0.49 (bootstrap 95% confidence interval 0.39 to 0.57; p < 0.001) interpreted as moderate success of blinding.

## Discussion

The results demonstrate that calf muscle stretching over a two-week period produces no statistically significant beneficial effects for foot pain and general foot health compared with not stretching. Although there was a trend for greater improvement in the stretching group in 'first-step' pain (by approximately 8 points on the 100 point Visual Analogue Scale) and foot function (by approximately 7 points on the 100 point Foot Health Status Questionnaire) these were also not statistically significant. In regards to foot function, no minimal important difference is known for the outcome and therefore the trial may have been underpowered to detect a statistically significant difference. Further, no differences were found in secondary outcomes of ankle range of motion and foot posture.

Participant characteristics in this trial were similar to samples in previous heel pain trials [13,29-31]. Participants were primarily middle-aged, overweight and spent the majority of the day on their feet. Likewise, participants also presented with relatively chronic symptoms.

The majority of adverse events in this trial were described by participants as short-lived and mild to moderate in intensity. Only one participant discontinued stretching due to an adverse event. Four events were due to increased pain while stretching, another four were due to calf pain and two due to a new pain in the lower limb. Participants noticed that the use of the wooden wedge placed increased pressure under the heel as the position of the foot on the wedge during the stretching procedure redistributed force away from the forefoot to the plantar heel region. This could have led to the four participants reporting increased pain while stretching. Caution should therefore be used in instructing participants to stretch their calf muscles by raising their forefoot from the ground (e.g. on a book or wedge similar to the one used in this trial). While we chose this form of stretching because it standardised the stretching technique across participants, alternative stretching methods that do not place increased pressure on the plantar heel (e.g. lowering the heel while the forefoot is on a step) may avoid such an adverse event.

The findings of this trial need to be viewed in light of some limitations. Firstly, the evidence from this trial is for one particular technique of stretching the calf muscle. Although participants reported being able to feel a stretch, no increase in ankle range of motion was found. Other stretching techniques such as lunges (dynamic stretching), proprioceptive neuromuscular facilitation, or using splints (e.g. night stretching splints) may yield other results. The stretching technique we utilised – using a wooden wedge – ensured a suitable stretch was applied in a relatively controlled manner. Secondly, the trial specifically examined the effect of stretching over a two-week period as a short-term treatment for plantar heel pain; generally the period a patient waits for the fabrication of a longer-term treatment such as foot orthoses [29]. It would be of interest to evaluate the effectiveness of regular stretching over a longer period to investigate whether the intervention has a long-term effect. This may obviate the need to institute more expensive long-term treatments such as foot orthoses; although the risk of a higher incidence of adverse events may not make this worthwhile.

This is the first randomised trial to examine the effect of calf stretching compared with no stretching for plantar heel pain. Two previous randomised trials have examined two different stretching techniques for plantar heel pain without inclusion of a non-stretching control group [17,18]. DiGiovanni et al [17] compared calf muscle stretching with plantar fascia tissue stretches over eight weeks. Both groups experienced reductions in pain, however the plantar fascia stretches were found to provide a statistically significant greater reduction in pain when compared to the calf muscle stretches. Porter et al [18] compared sustained 3 minute calf stretches with intermittent 20 second calf stretches for plantar heel pain and found no significant differences in improvement between groups. However, without a sham or no stretching control group, it is not possible to attribute the observed improvements in either trial to the stretching. In view of the favourable natural history of plantar heel pain participants in the trial may have improved irrespective of treatment due to the spontaneous resolution of the condition or as a result of the placebo or Hawthorne effects. Our trial clearly addresses this limitation and represents a more precise estimate of the true effect of calf muscle stretches for plantar heel pain.

## Conclusion

When used for the short-term treatment of plantar heel pain, stretching for two weeks provides no statistically significant improvements in 'first-step' pain, foot pain, foot function and general foot health compared with a control group. It was also associated with mild to moderate short-lived adverse events. Based upon our results a program of calf muscle stretching, similar to that conducted in this trial, is not recommended for plantar heel pain.

## Authors' contributions

JAR conceptualised the trial, undertook a literature search, designed the trial, collected and analysed the data, interpreted the results and wrote the paper. JAR is guarantor of the paper. KBL assisted with the conceptualisation and design of the trial, analysis of the data, interpretation of the results and writing of the paper. RB assisted with the conceptualisation and design of the trial and writing of the paper. CC assisted with the planning of the project and writing of the paper. All authors have read and approved the final manuscript.

## Pre-publication history

The pre-publication history for this paper can be accessed here:



## Figures and Tables

**Table 1:** Baseline characteristics of participants. Values are means (± standard deviation) unless stated otherwise.

**Variable**	**Stretching (n = 46)**	**Control (n = 46)**
Age in years	50.7 (± 11.8)	50.1 (± 11.0)
BMI in kg/m^2^	31.6 (± 5.8)	32.1 (± 6.5)
Self reported hours on feet	7.5 (± 3.5)	9.1 (± 3.7)
Period of symptoms in months – median (range)	13 (4 to 61)	13 (3 to 121)
No. of women (%)	31 (67.4)	25 (54.3)
Foot affected		
Right – No. (%)	15 (32.6)	17 (37.0)
Left – No. (%)	9 (19.6)	13 (28.3)
Both feet – No. (%)	22 (47.8)	16 (34.8)

**Table 2:** Primary outcomes at baseline and follow-up.

	**Group**			
**Primary outcome measures**	**Stretching – mean (SD)**	**Control – mean (SD)**	**Adjusted mean difference between groups (95% CI)**	**P-value**
'First-step' pain*				
Baseline	70.9 (± 23.0)	75.8 (± 19.1)		
Follow-up	51.1 (± 29.1)	62.5 (± 29.5)		
Mean change	-19.8 (± 26.0)	-13.2 (± 25.2)	-7.9 (-18.3 to 2.6)	0.138
Foot painΨ				
Baseline	34.0 (± 21.5)	31.7 (± 17.8)		
Follow-up	50.9 (± 23.1)	50.8 (± 26.4)		
Mean change	16.9 (± 20.4)	19.2 (± 21.6)	-1.6 (-10.1 to 6.9)	0.709
Foot functionΨ				
Baseline	56.3 (± 24.5)	58.2 (± 24.0)		
Follow-up	72.4 (± 23.6)	66.4 (± 26.2)		
Mean change	16.2 (± 19.5)	8.3 (± 18.5)	7.3 (-0.1 to 14.8)	0.052
General foot healthΨ				
Baseline	30.8 (± 21.7)	31.5 (± 24.4)		
Follow-up	37.3 (± 25.9)	43.3 (± 29.0)		
Mean change	6.5 (± 20.8)	11.7 (± 20.7)	-5.4 (-13.8 to 3.1)	0.211

* Measured using a Visual Analogue Scale (0 to 100 mm – 0 is no pain and 100 is the worst pain).

Ψ Measured using the Foot Health Status Questionnaire (0 to 100 – 0 is the worst foot health and 100 is the best foot health)

**Note**: The Foot Health Status Questionnaire is 'reverse-scored' compared to the Visual Analogue Scale. In the results above, the *negative *adjusted mean difference between groups in improvement for the comparison of 'first-step' pain favours stretching because it has lower pain. In comparison, a *positive *score for the Foot Health Status Questionnaire domains favours the stretching group

**Table 3:** Secondary outcomes at baseline and follow-up.

	**Group**			
**Secondary outcome measures**	**Stretching – mean (SD)**	**Control – mean (SD)**	**Adjusted mean difference between groups (95% CI)**	**P-value**
Right ankle dorsiflexion^§ ^in degrees				
Baseline	25.8 (± 5.6)	23.6 (± 5.5)		
Follow-up	25.6 (± 5.4)	24.4 (± 5.7)		
Mean change	0.2 (± 4.3)	-0.9 (± 3.0)	0.5 (-0.9 to 2.0)	0.470
Left ankle dorsiflexion^§ ^in degrees				
Baseline	25.8 (± 5.5)	24.0 (± 5.2)		
Follow-up	25.9 (± 5.7)	25.4 (± 6.2)		
Mean change	-0.1 (± 3.7)	-1.3 (± 3.9)	1.0 (-0.6 to 2.6)	0.216
Right Foot Posture†				
Baseline	3 (± 3)	2 (± 2)		
Follow-up	2 (± 3)	2 (± 2)		
Mean change	0 (± 2)	0 (± 1)	0 (-1 to 1)	0.993
Left Foot Posture*				
Baseline	3 (± 3)	3 (± 2)		
Follow-up	3 (± 3)	2 (± 2)		
Mean change	0 (± 2)	0 (± 2)	0 (-1 to 1)	0.841

§ Ankle range of motion (ROM) measured using the Lunge Test [20]

† The Foot Posture Index-6 has a score range of -12 to 12 with negative values for signs of foot supination and positive values for signs of foot pronation [21].

**Note**: In the results above, the *positive *adjusted mean difference between groups in improvement for the comparison of ankle dorsiflexion favours stretching because it has greater ankle dorsiflexion.
